# Distinct functional properties of two electrogenic isoforms of the SLC34 Na‐Pi cotransporter

**DOI:** 10.14814/phy2.14156

**Published:** 2019-07-24

**Authors:** Natsuki Mizutani, Yoshifumi Okochi, Yasushi Okamura

**Affiliations:** ^1^ Laboratory of Integrative Physiology Department of Physiology Graduate School of Medicine Osaka University Suita Osaka Japan; ^2^ Graduate School of Frontier Biosciences Osaka University Suita Osaka Japan

**Keywords:** inorganic phosphate, phosphatidylinositol 4, 5-bisphosphate, Phosphatidylinositol 4-phosphate, Type II Na-Pi cotransporter

## Abstract

Inorganic phosphate (P_i_) is crucial for proper cellular function in all organisms. In mammals, type II Na‐Pi cotransporters encoded by members of the *Slc34* gene family play major roles in the maintenance of P_i_ homeostasis. However, the molecular mechanisms regulating Na‐Pi cotransporter activity within the plasma membrane are largely unknown. In the present study, we used two approaches to examine the effect of changing plasma membrane phosphatidylinositol 4,5‐bisphosphate (PI(4,5)P_2_) levels on the activities of two electrogenic Na‐Pi cotransporters, NaPi‐IIa and NaPi‐IIb. To deplete plasma membrane PI(4,5)P_2_ in *Xenopus* oocytes, we utilized *Ciona intestinalis* voltage‐sensing phosphatase (Ci‐VSP), which dephosphorylates PI(4,5)P_2_ to phosphatidylinositol 4‐phosphate (PI(4)P). Upon activation of Ci‐VSP, NaPi‐IIb currents were significantly decreased, whereas NaPi‐IIa currents were unaffected. We also used the rapamycin‐inducible Pseudojanin (PJ) system to deplete both PI(4,5)P_2_ and PI(4)P from the plasma membrane of cultured Neuro 2a cells. Depletion of PI(4,5)P_2_ and PI(4)P using PJ significantly reduced NaPi‐IIb activity, but NaPi‐IIa activity was unaffected, which excluded the possibility that NaPi‐IIa is equally sensitive to PI(4,5)P_2_ and PI(4)P. These results indicate that NaPi‐IIb activity is regulated by PI(4,5)P_2_, whereas NaPi‐IIa is not sensitive to either PI(4,5)P_2_ or PI(4)P. In addition, patch clamp recording of NaPi‐IIa and NaPi‐IIb currents in cultured mammalian cells enabled kinetic analysis with higher temporal resolution, revealing their distinct kinetic properties.

## Introduction

Inorganic phosphate (P_i_) is essential for a variety of cell activities, including bioenergetics and cell signaling. Phosphate is also an essential constituent of biological membranes and bone matrices, which are composed mainly of calcium phosphates. Dietary P_i_ is absorbed in the small intestine and circulates in the blood, where its concentration is controlled mainly through reabsorption in the kidney. Absorption of P_i_ in both the intestine and kidney is mediated primarily by type II Na‐Pi cotransporters (or SLC34 proteins), which consist of three members, NaPi‐IIa (SLC34A1), NaPi‐IIb (SLC34A2), and NaPi‐IIc (SLC34A3), showing different patterns of expression (Wagner et al. 2014): NaPi‐IIa and NaPi‐IIc are localized specifically in the apical brush border membrane of renal proximal tubule cells, whereas NaPi‐IIb is found in many tissues, including the luminal brush border membrane of the small intestine and alveolar type II epithelial cells (Traebert et al. 1999; Wagner et al. 2014). All three forms transport one divalent P_i_ (HPO_4_^2−^) coupled with multiple Na^+^ per transport cycle, but with different P_i_:Na^+^ stoichiometries: NaPi‐IIa and NaPi‐IIb show a 1:3 stoichiometry (electrogenic), whereas NaPi‐IIc exhibits a 1:2 stoichiometry (electroneutral) (Forster 2019). Studies of knockout mice and human disease have shown the importance of these transporters at the whole body level. Mice lacking NaPi‐IIa exhibit hypophosphatemia and hyperphosphaturia (Beck et al. 1998), while those lacking NaPi‐IIb die in utero (Shibasaki et al. 2009). Human NaPi‐IIc mutations cause hereditary hypophosphatemic rickets with hypercalciuria (Bergwitz et al. 2006).

P_i_ absorption mediated by Na‐Pi cotransporters is known to be regulated by hormones such as parathyroid hormone (PTH), fibroblast growth factor 23 (FGF23), and 1,25‐(OH)_2_‐Vitamin D_3_, which change the number of these transporters at the cell surface. PTH facilitates endocytosis of NaPi‐IIa and NaPi‐IIc in kidney, reducing reabsorption of P_i_ (Segawa et al. 2007; Picard et al. 2010). 1,25‐(OH)_2_‐Vitamin D_3_, which stimulates calcium uptake in the small intestine, increases the number of NaPi‐IIb in the brush border membrane of this tissue (Hattenhauer et al. 1999). These cotransporter activities are also regulated by membrane potential (Forster et al. 1998; Hilfiker et al. 1998) and extracellular pH (de la Horra et al. 2000). However, other regulation mechanisms at the plasma membrane remain to be explored.

The activities of many ion channels and some transporters are reportedly regulated by phosphatidylinositol 4,5‐bisphosphate (PI(4,5)P_2_) (Suh and Hille 2008), which is the most common phosphoinositide in the plasma membrane and accounts for 1% of phospholipid (Mclaughlin et al. 2002). PI(4,5)P_2_ can be hydrolyzed by receptor‐activated phospholipase C (PLC) to produce inositol trisphosphate (IP_3_) and diacylglycerol (DAG) (Rhee and Choi 1992). PI(4,5)P_2_‐sensitive ion channel activities are regulated through stimulation of G_q_‐coupled receptors, which in turn mediate PLC‐catalyzed PI(4,5)P_2_ hydrolysis (Hille et al. 2015). Such regulation is well characterized in cardiac cells and neurons (Cho et al. 2005; Morita et al. 2006). In neurons, for example, KCNQ2/3 activity is regulated by G_q_‐coupled receptors that inhibit channel activity, thereby enhancing neurotransmitter‐induced excitation (Hughes et al. 2007).

Over the last decade, knowledge about the role of PI(4,5)P_2_ in the regulation of various types of channels has been obtained through experiments using several molecular tools to manipulate phosphoinositide levels (Balla 2013; Hille et al. 2015; Okamura et al. 2018). Voltage‐sensing phosphatase (VSP) dephosphorylates PI(4,5)P_2_ upon membrane depolarization (Murata and Okamura 2007; Iwasaki et al. 2008; Okamura et al. 2018), thereby inducing its rapid and reversible depletion. Rapamycin‐inducible enzymes are another tool for manipulating phosphoinositide levels. For example, Pseudojanin (PJ) is a chimeric protein that exhibits lipid 5‐phosphatase and 4‐phosphatase activities when complexed with a plasma membrane‐associated anchor protein in the presence of rapamycin (Hammond et al. 2012). In contrast to the numerous examples of PI(4,5)P_2_‐dependent ion channel regulation, there have been fewer studies of the PI(4,5)P_2_ sensitivity of transporter proteins (Dickson and Hille 2019). So far, six types of transporters have been shown to be regulated by PI(4,5)P_2_: the Na^+^‐Ca^2+^ exchanger (NCX1) (Hilgemann and Ball 1996; Yaradanakul et al. 2007), Na^+^/bicarbonate cotransporter (NBCe1) (Wu et al. 2009; Thornell et al. 2012; Thornell and Bevensee 2015), Na^+^/H^+^ exchanger (NHE1) (Aharonovitz et al. 2000), plasma membrane Ca^2+^‐ATPase (PMCA1) (Choquetteayc et al. 1984), serotonin transporter (SERT) (Buchmayer et al. 2013), and dopamine transporter (DAT) (Hamilton et al. 2014). In the present study, we examined the dependence of two types of mouse (m) Na‐Pi cotransporters, mNaPi‐IIa and mNaPi‐IIb, on PI(4,5)P_2_. We found that mNaPi‐IIa activity is unaffected by PI(4,5)P_2_ depletion using VSP and rapamycin‐inducible PJ, whereas mNaPi‐IIb activity is inhibited by PI(4,5)P_2_ depletion. These results indicate that mNaPi‐IIa and mNaPi‐IIb have different sensitivities to PI(4,5)P_2_. Furthermore, we measured mNaPi‐IIa and mNaPi‐IIb currents in cultured mammalian cells, Neuro 2a, using the whole‐cell patch clamp technique and revealed their distinct kinetic properties.

## Methods

### Ethics approval

Experiments using *Xenopus laevis* were performed in accordance with the guidelines of the Animal Care and Use Committee of the Osaka University Graduate School of Medicine.

### Molecular biology

Wild type (WT) mNaPi‐IIa (NM_011392.2) and mNaPi‐IIb (NM_011402.3) cDNAs were cloned from mouse kidney total cDNA using RT‐PCR and subcloned into a custom modified version of the pcs2+ expression vector (Rupp et al. 1994). For mammalian cell expression, mNaPi‐IIa with FLAG fused at its N‐terminus and mNaPi‐IIb were subcloned into pIRES2‐EGFP vector. Rat Kv7.2 (rKCNQ2) and rat Kv7.3 (rKCNQ3), both in pGEM‐HE, was a kind gift from Drs. David McKinnon and Koichi Nakajo. The cDNA encoding the *α*‐subunit of human cardiac voltage‐gated sodium channel, SCN5A (hH1), in pcDNA1 was a kind gift from Dr. Mohamed Chahine. PJ (Addgene ID: 37999) and Lyn_11_‐FRB‐CFP (Addgene ID: 38003) were obtained from Addgene. PJ without monomeric red fluorescent protein (mRFP) (named PJ mRFP (‐)) was subcloned into pcs2+. Cyan fluorescent protein (CFP) was replaced with mCherry, and Lyn_11_‐FRB‐mCherry was subcloned into pcDNA3.1. PH_PLC*δ*1_‐GFP was a kind gift from Drs. Makoto Takano and Nobuyuki Uozumi. GFP was replaced with mCherry, after which PH_PLC*δ*1_‐mCherry was subcloned into pcDNA3.1. GFP‐P4M‐SidMx1 (Addgene ID: 51469) was obtained from Addgene.

### Preparation of oocytes

*Xenopus laevis* oocytes were collected from frogs anesthetized in water containing 0.2% ethyl 3‐aminobenzoate methanesulfonate salt (Sigma‐Aldrich, USA). The isolated oocytes were defolliculated by treatment with type I collagenase (1.0 mg/ml, Sigma‐Aldrich) in ND96 solution (96 mmol/L NaCl, 2 mmol/L KCl, 1.8 mmol/L CaCl_2_, 1 mmol/L MgCl_2_, 5 mmol/L HEPES, 0.1 mg/ml gentamycin, 5 mmol/L Sodium Pyruvate, pH 7.5 [adjusted with NaOH]) and then injected with 50 nl of cRNA. cRNAs were synthesized from linearized plasmid DNA using a mMESSAGE mMACHINE transcription kit (Thermo Fisher Scientific, USA). The oocytes were incubated for 1.5–2 days at 18°C in ND96.

### Two‐electrode voltage clamp technique

**Figure 1:**
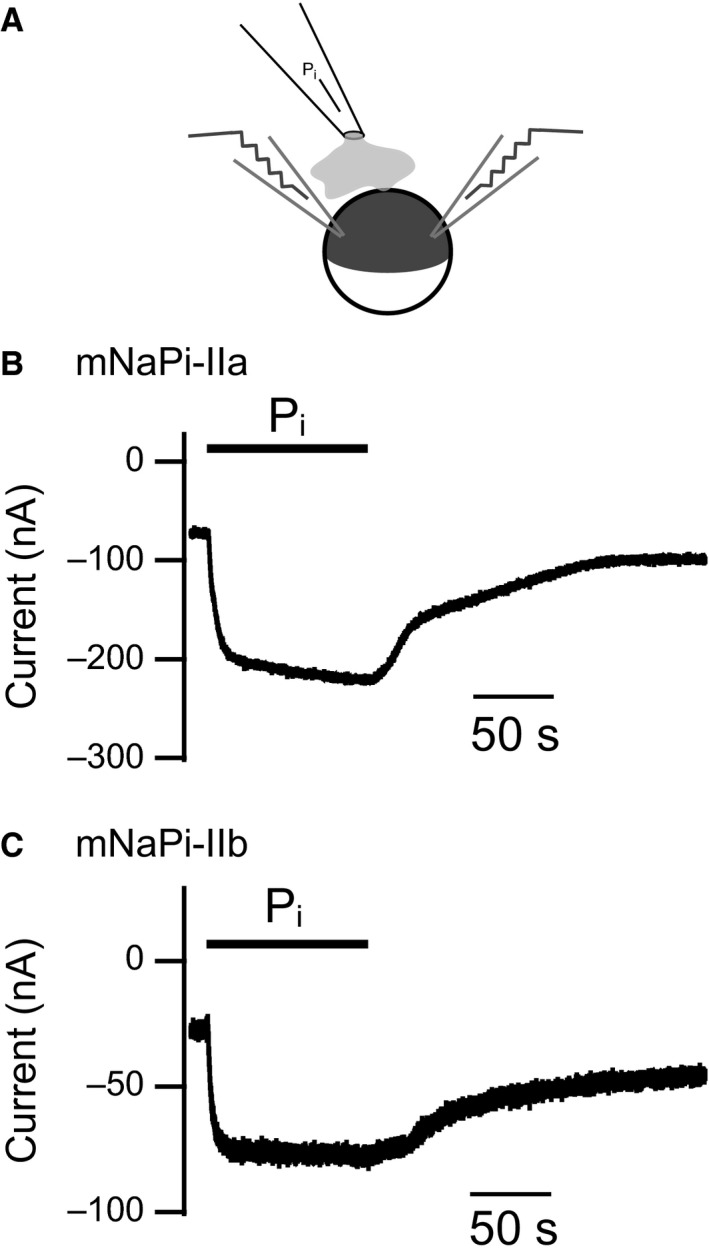
P_i_‐induced NaPi‐IIa and NaPi‐IIb currents in *Xenopus* oocytes. (A) schematic representation of the puff application system for TEVC recording. The oocytes were placed just under the tip of the puff pipette. B–C, traces of representative currents generated by mNaPi‐IIa (B) and mNaPi‐IIb (C) expressed in *Xenopus* oocytes. Oocytes were bathed in Ca^2+^‐free ND100 solution and clamped at ‐60 mV. Thick bars show the period of application of 10 mmol/L P_i_ solution.

Electrical recordings were made using two‐electrode voltage clamp (TEVC) carried out with an OC‐725C amplifier (Warner Instruments, USA). Output signals filtered at 1 kHz were digitized with an AD/DA converter (InstruTECH LIH 8 + 8; HEKA Elektronik, Germany) and analyzed using PatchMaster software (HEKA Elektronik). The resistance of the glass pipettes was 0.1–1.0 MΩ after filling with 2 mol/L KOAc and 1 mol/L KCl solution. The oocytes were placed in a bath chamber (volume, 1.0 ml) connected to an original gravity perfusion system. The oocytes were initially voltage clamped at −60 mV while being perfused with Ca^2+^‐free ND100 solution (100 mmol/L NaCl, 2 mmol/L KCl, 5 mmol/L MgCl_2_, 5 mmol/L HEPES, pH 7.6 [adjusted with NaOH]) at the rate of 1.02 ml/min. 100 mmol/L NaH_2_PO_4_/Na_2_HPO_4_ solution (pH 7.4) was diluted with Ca^2+^‐free ND100 solution to make following 1 and 10 mmol/L P_i_ solution. Once the current reached a steady state, the solution was changed to 1 mmol/L P_i_ solution (99 mmol/L NaCl, 1 mmol/L NaH_2_PO_4_/Na_2_HPO_4_, 2 mmol/L KCl, 5 mmol/L MgCl_2_, 5 mmol/L HEPES, pH 7.6). In the experiments summarized in Figure 1, 10 mmol/L P_i_ solution (90 mmol/L NaCl, 10 mmol/L NaH_2_PO_4_/Na_2_HPO_4_, 1.8 mmol/L KCl, 4.5 mmol/L MgCl_2_, 4.5 mmol/L HEPES, pH 7.6) was administered to the oocytes at a rate of 25.0 ml/h using the puff application system described in Figure 1A. To prevent P_i_ solution flowing out from the tip of the puff pipette before application, the tip was positioned such that it was not in contact with the surface of the bath solution.

In the experiments using Ci‐VSP, a depolarization pulse to +50 mV was applied for 10 sec to activate Ci‐VSP and to reduce PI(4,5)P_2_ levels in the plasma membrane. To check the plasma membrane PI(4,5)P_2_ level, rKCNQ2/3 currents were measured by following protocol: a step pulse to −10 mV was applied for 200 msec, 5 sec before and 5, 25, and 55 sec after Ci‐VSP activation. Holding potential was set to −60 mV which activates little rKCNQ2/3 currents (Zhang et al. 2003).

### Cell culture and transfection

Human embryonic kidney 293T (HEK293T) cells and undifferentiated mouse neuroblastoma (Neuro 2a) cells were cultured in Dulbecco's Modified Eagle's Medium (FUJIFILM Wako Pure Chemical Corporation, Japan) supplemented with 10% fetal bovine serum (Biowest, France) and penicillin/streptomycin (10 *μ*g/ml, nacalai tesque, Japan) at 37°C in an incubator under a 5% CO_2_/95% air atmosphere. cDNAs in mammalian cell expression vectors were transfected using Polyethylenimine (PEI) Max reagent (Polysciences, Inc., USA). After transfection for 5–6 h, the medium containing the mixture was replaced with new medium. For whole‐cell electrophysiological analysis of their PI(4,5)P_2_ or PI(4)P dependence, mNaPi‐IIa or mNaPi‐IIb was coexpressed with PJ mRFP (‐) and Lyn_11_‐FRB‐mCherry. For confocal fluorescence imaging, PJ mRFP (‐) was coexpressed with Lyn_11_‐FRB‐CFP and the mCherry‐fused pleckstrin homology domain (PH) of PLC*δ*1 (PH_PLC*δ*1_‐mCherry) or Lyn_11_‐FRB‐mCherry and GFP‐P4M‐SidMx1.

### Whole‐cell electrophysiology

Following transfection, cells were incubated for 16–18 h and then reseeded into poly‐L‐lysine (Sigma‐Aldrich)‐coated dishes. Electrophysiological recordings were made 3–10 h after reseeding. Whole‐cell patch clamp recordings were made using an EPC9 amplifier (HEKA Elektronik) and analyzed using PatchMaster software (HEKA Elektronik). Patch pipettes were pulled from borosilicate glass (Drummond Scientific Company, USA), and their resistance was 2–8 MΩ after filling with pipette solution. The pipette solution (110 Cs^+^ solution) contained 110 mmol/L CsMeSO_3_, 10 mmol/L NaCl, 1 mmol/L CaCl_2_, 2 mmol/L MgCl_2_, 10 mmol/L HEPES, 10 mmol/L TEACl, 10 mmol/L EGTA, pH 7.2 (adjusted with CsOH). The bath solution (140 NaCl solution) contained 140 mmol/L NaCl, 3 mmol/L KCl, 2 mmol/L CaCl_2_, 1 mmol/L MgCl_2_, 10 mmol/L HEPES, 1 mmol/L Glucose, pH 7.4 (adjusted with NaOH). HEK293T cells expressing SCN5A (hH1) and Neuro 2a cells expressing mNaPi‐IIa or mNaPi‐IIb were voltage clamped at −80 mV and −60 mV, respectively, and all currents were recorded at room temperature (22–26°C). During current recording, a rapid perfusion system (DAD‐VM; ALA Scientific Instruments, USA) was used to apply solutions. The flow rate was 42.0 *μ*l/min. In the experiments summarized in Figure 4B and D, Na^+^‐free solution containing 140 mmol/L NMDG, 3 mmol/L KCl, 2 mmol/L CaCl_2_, 1 mmol/L MgCl_2_, 10 mmol/L HEPES, 1 mmol/L Glucose, pH 7.4 (adjusted with HCl) was perfused to confirm a P_i_‐independent basal inward current and the speed of perfusion, respectively. To measure transporter currents, 5 mmol/L P_i_ in 140 NaCl solution (133 mmol/L NaCl, 5 mmol/L NaH_2_PO_4_/Na_2_HPO_4_, 3 mmol/L KCl, 2 mmol/L CaCl_2_, 1 mmol/L MgCl_2_, 9.5 mmol/L HEPES, 1 mmol/L Glucose, pH 7.4) was perfused. In experiments using the PJ system, 1 *μ*mol/L rapamycin (FUJIFILM Wako Pure Chemical Corporation) (1 mmol/L stock in DMSO) in the 5 mmol/L P_i_ solution was perfused to recruit PJ to the plasma membrane region.

### Confocal fluorescence imaging

Confocal fluorescence imaging was performed using an inverted LSM 710 confocal microscope (Carl Zeiss, Germany) at room temperature (22–25°C). Transfected cells were reseeded onto poly‐L‐lysine‐coated coverslips (Matsunami, Japan). Each coverslip was moved into the chamber for imaging analysis, which was filled with the 140 NaCl solution. Cells were scanned every 2 sec for 60 cycles. Between the 10th and 11th scan, 10 *μ*mol/L rapamycin in the 140 NaCl solution was added to the chamber; the final rapamycin concentration was estimated to be 1 *μ*mol/L. Acquired data were analyzed by using ImageJ software (NIH, USA).

### Data analysis

Data are presented as the mean ± standard error of the mean (SEM). All data were analyzed using PatchMaster and Igor Pro (WaveMetrics Inc., USA) software. Statistical comparisons were made using Microsoft Excel 2016 (Microsoft, USA). Values of *P *<* *0.05 were considered statistically significant.

## Results

### P_i_‐induced currents produced by two types of electrogenic Na‐Pi cotransporters in a *Xenopus* oocyte expression system

As in earlier reports (Hartmann et al. 1995; Hilfiker et al. 1998), we first measured the electrogenic transport activities of mNaPi‐IIa and mNaPi‐IIb using TEVC with a *Xenopus* oocyte expression system. To apply P_i_ solution more rapidly than a gravity perfusion system, we utilized a puff application system with a syringe pump (Fig. 1A). In oocytes expressing mNaPi‐IIa, an increase in the inward current was observed after application of 10 mmol/L P_i_ solution, as was previously reported (Hartmann et al. 1995) (Fig. 1B). After washing out the P_i_, the current gradually returned to the initial basal level. A similar inward current induced by application of external P_i_ was also observed with mNaPi‐IIb (Fig. 1C), as was previously reported (Hilfiker et al. 1998).

### NaPi‐IIa activity is unaffected by PI(4,5)P_2_ depletion by Ci‐VSP

**Figure 2:**
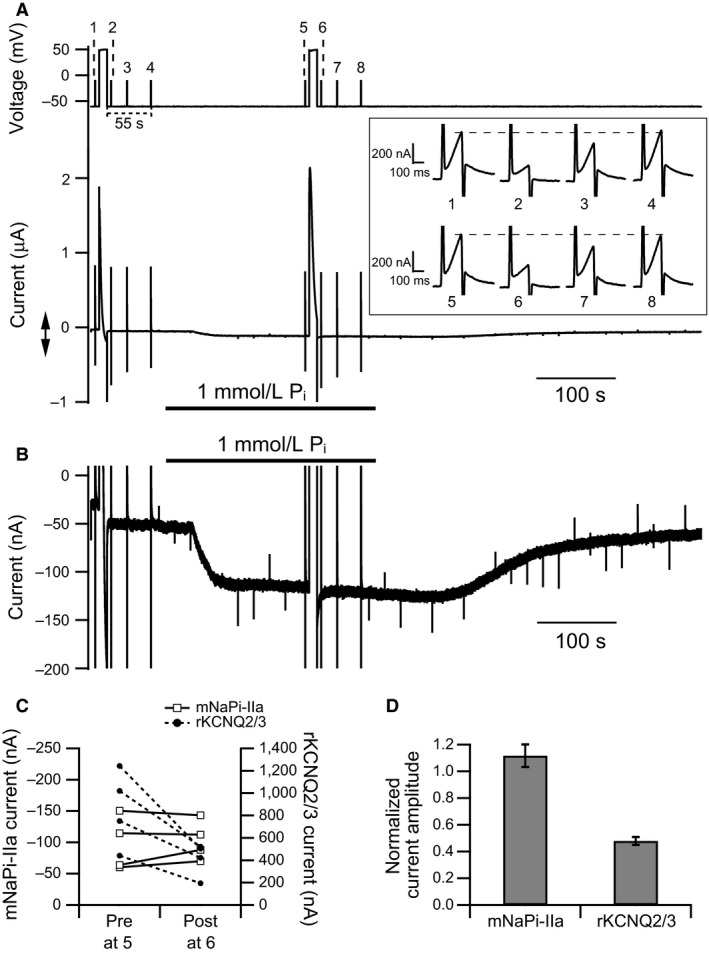
Ci‐VSP‐induced PI(4,5)P_2_ depletion has no effect on NaPi‐IIa but inhibits KCNQ2/3 activity. (A) pulse protocol (upper) and membrane currents (lower) in an oocyte coexpressing mNaPi‐IIa, rKCNQ2, rKCNQ3, and Ci‐VSP. rKCNQ2 and rKCNQ3 form heterotetrameric rKCNQ2/3 channels, which carry delayed‐rectifier voltage‐gated potassium currents sensitive to PI(4,5)P_2_. To activate Ci‐VSP, oocytes were depolarized to +50 mV for 10 sec twice. The first 10‐sec depolarization was applied before application of P_i_ in the bath solution and the second after application of P_i_. To measure rKCNQ2/3 currents, depolarization steps to −10 mV were applied for 200 msec, 5 sec before and 5, 25, and 55 sec after each 10‐sec depolarization. The inset shows rKCNQ2/3 currents recorded to monitor the PI(4,5)P_2_ levels on an enlarged timescale at times 1–4 and 5–8 of the pulse protocol. Membrane potential was maintained at −60 mV throughout the recording. (B) mNaPi‐IIa current trace shown on an enlarged current scale indicated by the double arrow in A. (C) changes in mNaPi‐IIa (open squares and solid line) and rKCNQ2/3 (filled circles and dashed line) current amplitudes in response to PI(4,5)P_2_ depletion induced by Ci‐VSP activation. Left axis shows the scale for the mNaPi‐IIa current and the right axis shows the scale for the rKCNQ2/3 current. (D) summary of the effect of Ci‐VSP‐induced PI(4,5)P_2_ depletion on mNaPi‐IIa and rKCNQ2/3 currents (*n* = 4). Current amplitudes after the 10‐sec depolarization were normalized to the amplitudes before depolarization. Data are means ± SEM.

We tested the sensitivity of mNaPi‐IIa to PI(4,5)P_2_ by depleting the phosphoinositide using Ci‐VSP, which dephosphorylates PI(4,5)P_2_ upon depolarization of the membrane potential (Murata and Okamura 2007; Okamura et al. 2018). The extent of the reduction in PI(4,5)P_2_ was monitored electrophysiologically by coexpressing rKCNQ2/3 ion channels. mNaPi‐IIa and rKCNQ2/3 currents were recorded from single oocytes to evaluate the relationship between the PI(4,5)P_2_ level and mNaPi‐IIa current (Fig. 2A and B). When the cell membrane was depolarized to +50 mV for 10 sec after the mNaPi‐IIa current reached a steady state (Fig. 2A and B), there was no change in the mNaPi‐IIa current (Pre: 97 ± 19 nA, Post: 103 ± 14 nA, *n* = 4, paired *t*‐test: *P* = 0.44, ratio: 1.12 ± 0.08) (Fig. 2C and D). By contrast, rKCNQ2/3 currents were markedly decreased by the depolarization (Fig. 2A, trace 5 and 6 in the inset) (Pre: 864 ± 300 nA, Post: 410 ± 130 nA, *n* = 4, paired *t*‐test: *P* = 0.025, ratio: 0.48 ± 0.03) (Fig. 2C and D), indicating PI(4,5)P_2_ was depleted during that period. We also confirmed that within 55 sec after terminating the 10‐sec depolarization (shown in upper panel of Fig. 2A), the rKCNQ2/3 current had recovered to the predepolarization level (Fig. 2A, trace 1–4 and 5–8 in the inset), indicating that PI(4,5)P_2_ levels are able to completely recover following repolarization of the membrane. These results show that mNaPi‐IIa is insensitive to PI(4,5)P_2_, in the range of PI(4,5)P_2_ reductions attained through activation of Ci‐VSP.

### PI(4,5)P_2_ depletion by Ci‐VSP inhibits NaPi‐IIb activity

**Figure 3:**
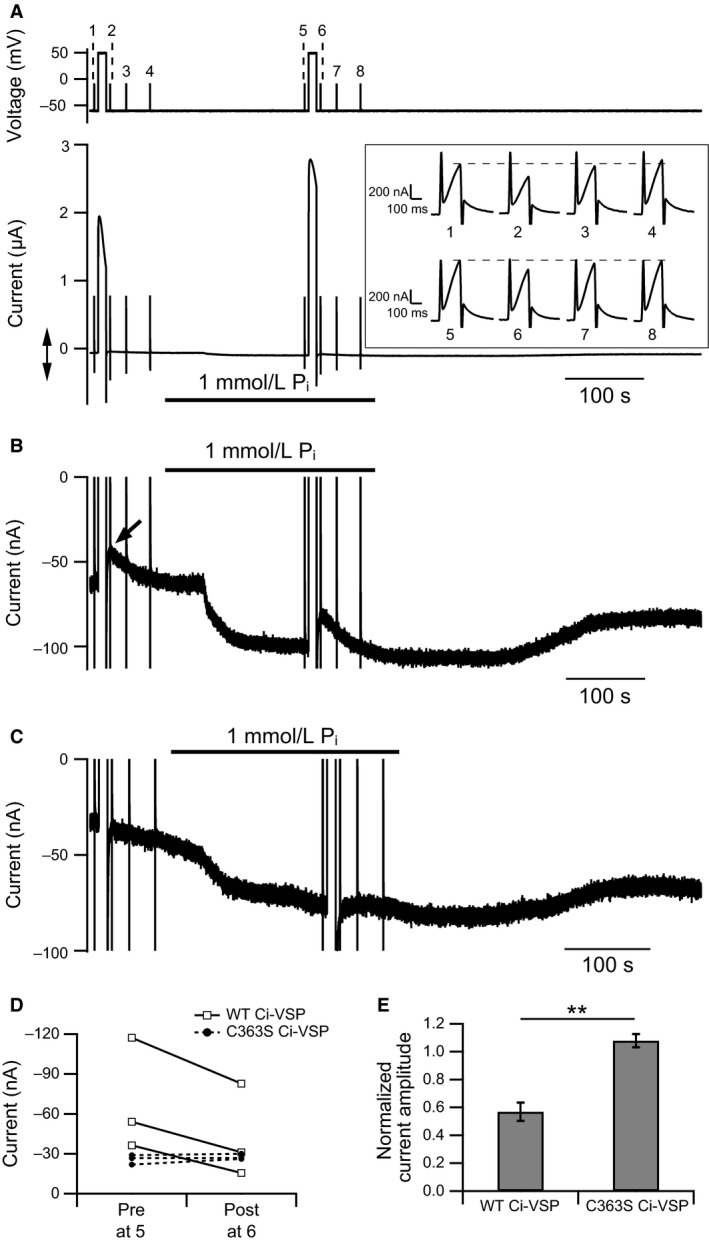
Effect of Ci‐VSP‐induced PI(4,5)P_2_ depletion on NaPi‐IIb activity. (A) Pulse protocol (upper) and membrane currents (lower) in an oocyte coexpressing mNaPi‐IIb, rKCNQ2, rKCNQ3, and WT Ci‐VSP. The protocol is the same as in Fig. 2A. The inset shows rKCNQ2/3 currents recorded to monitor the PI(4,5)P_2_ levels on an enlarged timescale at times 1–4 and 5–8 of the pulse protocol. (B) mNaPi‐IIb current trace on an enlarged current scale shown by the double arrow in A. An arrow points to the current decrease after the first 10‐sec depolarization in the absence of P_i_. (C) Representative mNaPi‐IIb current trace on an enlarged current scale obtained from an oocyte coexpressing with rKCNQ2, rKCNQ3, and C363S Ci‐VSP. The protocol is the same as in A. (D) Changes in the amplitudes of currents generated by mNaPi‐IIb in cells coexpressed WT Ci‐VSP (open squares and solid line) or C363S Ci‐VSP (filled circles and dashed line). (E) Summary of the effect of Ci‐VSP‐induced PI(4,5)P_2_ depletion on mNaPi‐IIb currents. Current amplitude after the 10‐sec depolarization was normalized to that before depolarization. Normalized current amplitudes were compared using Student's *t*‐test (*n* = 3 for both WT and C363S Ci‐VSP, means ± SEM, **P <* *0.01).

We examined the sensitivity of mNaPi‐IIb to PI(4,5)P_2_ using the same protocol used for mNaPi‐IIa (Fig. 3A). In contrast to mNaPi‐IIa currents, the 10‐sec depolarization led to reductions in the mNaPi‐IIb current (Fig. 3B). Immediately after the depolarization, mNaPi‐IIb currents were significantly smaller than before it (Pre: 69 ± 20 nA, Post: 43 ± 17 nA, *n* = 3, paired *t*‐test: *P* = 0.025, ratio: 0.57 ± 0.07) (Fig. 3D). Within 55 sec after membrane repolarization, the currents had recovered to the predepolarization amplitude (Fig. 3B). Recovery of the rKCNQ2/3 currents was also observed (Fig. 3A, trace 5–8 in the inset). To verify that it was PI(4,5)P_2_ depletion by Ci‐VSP that inhibited the mNaPi‐IIb activity, we assessed mNaPi‐IIb activity using the same protocol with a Ci‐VSP C363S mutant in which a cysteine in the phosphatase active center is substituted with a serine, eliminating its enzymatic activity (Murata et al. 2005). Using the mutated Ci‐VSP, neither the mNaPi‐IIb nor the rKCNQ2/3 current was affected by the 10sec depolarization (for mNaPi‐IIb, Pre: 26 ± 2 nA, Post: 28 ± 1 nA, *n* = 3, paired *t*‐test: *P* = 0.28, ratio: 1.08 ± 0.05) (Fig. 3C and D). Following the depolarization, mNaPi‐IIb current amplitudes were significantly smaller with WT Ci‐VSP than with the C363S mutant (Fig. 3E). These results indicate that mNaPi‐IIb is sensitive to the membrane PI(4,5)P_2_ level, which is diminished by Ci‐VSP activity.

Interestingly, even in the absence of P_i_, holding currents decreased after the 10‐sec depolarization (Fig. 3B arrow) and then gradually returned to the level before the depolarization as found in rKCNQ2/3 currents (Fig. 3A, trace 1‐4 in the inset). No changes in the holding current were observed when the Ci‐VSP C363S mutant was expressed (Fig. 3C). Previous studies reported that in the absence of P_i_, holding current contains a baseline current carried by Na^+^ in oocytes heterologously expressing mouse NaPi‐IIa, rat NaPi‐IIa, and flounder NaPi‐IIb (Forster et al. 2002; Andrini et al. 2008). These led us to speculate that the holding currents in our experiments also contained such baseline current derived from P_i_‐independent basal transporter activity (hereafter, thus called the “P_i_‐independent basal inward current”). We conclude that both the P_i_‐independent basal inward current and currents linked with P_i_ transport by mNaPi‐IIb are sensitive to PI(4,5)P_2_ depletion.

### NaPi‐IIa and NaPi‐IIb currents heterologously expressed in Neuro 2a cells

Ci‐VSP‐mediated dephosphorylation of PI(4,5)P_2_ leads to production of PI(4)P (Iwasaki et al. 2008). Therefore, a possible explanation for the lack of change in the amplitude of mNaPi‐IIa currents with activation of Ci‐VSP may be that mNaPi‐IIa is sensitive to both PI(4,5)P_2_ and PI(4)P. To test this possibility, we made use of the engineered chimeric lipid phosphatase PJ, which has both 5‐phosphatase and 4‐phosphatase activities (Hammond et al. 2012). In this system, recruitment of PJ to the plasma membrane can be readily induced by adding rapamycin to the bath solution. Upon binding of rapamycin to PJ, the enzyme is recruited to the plasma membrane through linkage to a scaffold protein (Lyn_11_‐FRB) localized at the membrane, which in turn leads to depletion of both PI(4,5)P_2_ and PI(4)P (Hammond et al. 2012). However, in our pilot study using PJ in *Xenopus* oocytes, we were unable to induce robust phosphatase activity. We therefore decided to use a mammalian cell expression system.

**Figure 4:**
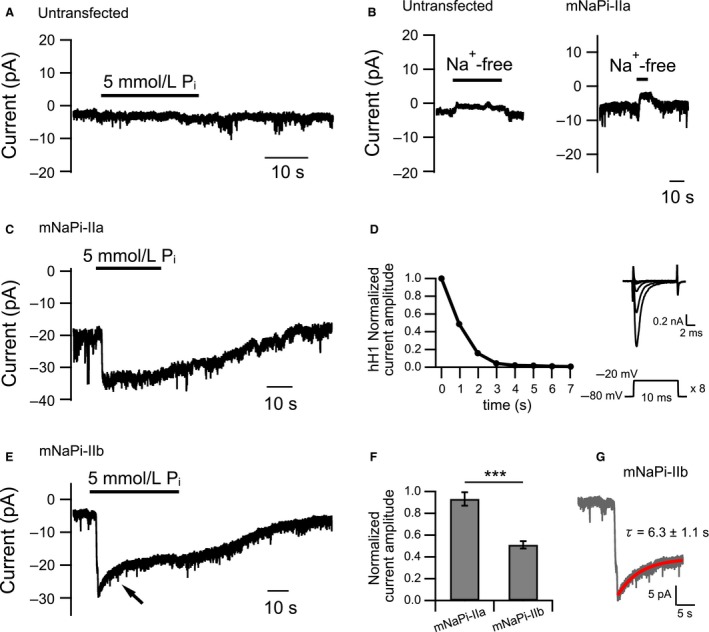
P_i_‐induced NaPi‐IIa and NaPi‐IIb currents in Neuro 2a cells. (A) Representative current trace recorded from an untransfected Neuro 2a cell. Neuro 2a cells exhibit no endogenous current in response to application of 5 mmol/L P_i_ solution. Cells were bathed in 140 mmol/L NaCl solution. (B) Representative current traces recorded from an untransfected (left) and a mNaPi‐IIa‐expressing (right) Neuro 2a cell bathed in 140 mmol/L NaCl solution. Na^+^‐free solution was applied during the period indicated by the thick bar. (C) Representative current trace recorded from a mNaPi‐IIa‐expressing Neuro 2a cell. An inward current was elicited upon application of 5 mmol/L P_i_ solution. (D) Left: representative time course of SCN5A (hH1) current decline upon rapid perfusion of Na^+^‐free solution by using an ALA Scientific perfusion system. Right: current traces recorded from a HEK293T cell expressing SCN5A (hH1) (upper) and the pulse protocol (lower). Eight current traces recorded at different times during the solution exchange are superimposed. Currents were induced by 10‐msec step pulses to ‐20 mV every 1 sec after starting perfusion at 0 sec; the pulse was repeated eight times. The holding potential was −80 mV. (E) Representative current trace recorded from a mNaPi‐IIb‐expressing Neuro 2a cell. 5 mmol/L P_i_ solution was applied during the period indicated by the thick bar. An arrow points to the current decay. (F) Relative amplitudes of currents recorded 19 sec after reaching its maximal amplitude. Currents were normalized to the maximal amplitude. Normalized amplitudes were compared using Student's *t*‐test (means ± SEM, ***P <* *0.001). (G) The trace in E shown on an enlarged timescale (gray trace). The current decay was fitted by a single‐exponential equation (red line). The time constant is the mean ± SEM (*n* = 8). In A, B, C, and E, membrane potential was clamped to −60 mV.

The whole‐cell patch clamp recording of type II Na‐Pi cotransporters from cultured mammalian cells has not been reported. We initially chose HEK293T cells as a heterologous expression system, but an inward current induced by P_i_ was occasionally recorded even in untransfected cells possibly due to some endogeneous P_i_‐sensitive channel or transporter. We next tried Neuro 2a cells. Unlike with HEK293T cells, no inward currents were recorded from untransfected Neuro 2a cells (*n* = 9) (Fig. 4A). When we transfected the cells with mNaPi‐IIa‐pIRES2‐EGFP plasmid and measured the currents from GFP‐positive cells, we were able to record inward transporter currents after 5 mmol/L P_i_ solution was perfused using an ALA Scientific rapid perfusion system (Fig. 4C). The current recovery to baseline after washing out the external P_i_ was slow. We also observed a P_i_‐independent basal inward current (Fig. 4C), as was previously reported in oocyte experiments (Forster et al. 2002; Andrini et al. 2008). This basal inward current decreased in Na^+^‐free solution (Fig. 4B), consistent with the idea that it is carried by Na^+^. By measuring currents through SCN5A (hH1), a human cardiac voltage‐gated Na^+^ channel, with distinct external Na^+^ concentrations, we estimated the time needed for replacement of the external solutions to be within 5 sec (Fig. 4D). This suggests that the slow current recovery reflects an innate property of mNaPi‐IIa, not a slow exchange rate of external P_i_ in our recording system.

Of note, the kinetics of P_i_‐induced currents of mNaPi‐IIb differed from those of mNaPi‐IIa. In the presence of P_i_, mNaPi‐IIb exhibited marked current decay after the current reached its maximum (Fig. 4E arrow). No such current decay was seen in studies with *Xenopus* oocytes (Hilfiker et al. 1998). The relative mNaPi‐IIb current amplitude 19 sec after reaching the peak was significantly smaller than the mNaPi‐IIa current (mNaPi‐IIb: 0.51 ± 0.03, *n* = 8 *vs*. mNaPi‐IIa: 0.93 ± 0.06, *n* = 7) (Fig. 4F). The decay phase of the mNaPi‐IIb current was fitted by a single‐exponential function, and its time constant (*τ*) was 6.3 ± 1.1 sec (Fig. 4G). Like mNaPi‐IIa (Fig. 4C), mNaPi‐IIb exhibited a slow recovery after P_i_ washout (Fig. 4E). A P_i_‐independent basal inward current decreased in Na^+^‐free solution as with mNaPi‐IIa, consistent with the idea that it is also carried by Na^+^.

### Depletion of PI(4,5)P_2_ and PI(4)P using PJ does not affect NaPi‐IIa activity

**Figure 5:**
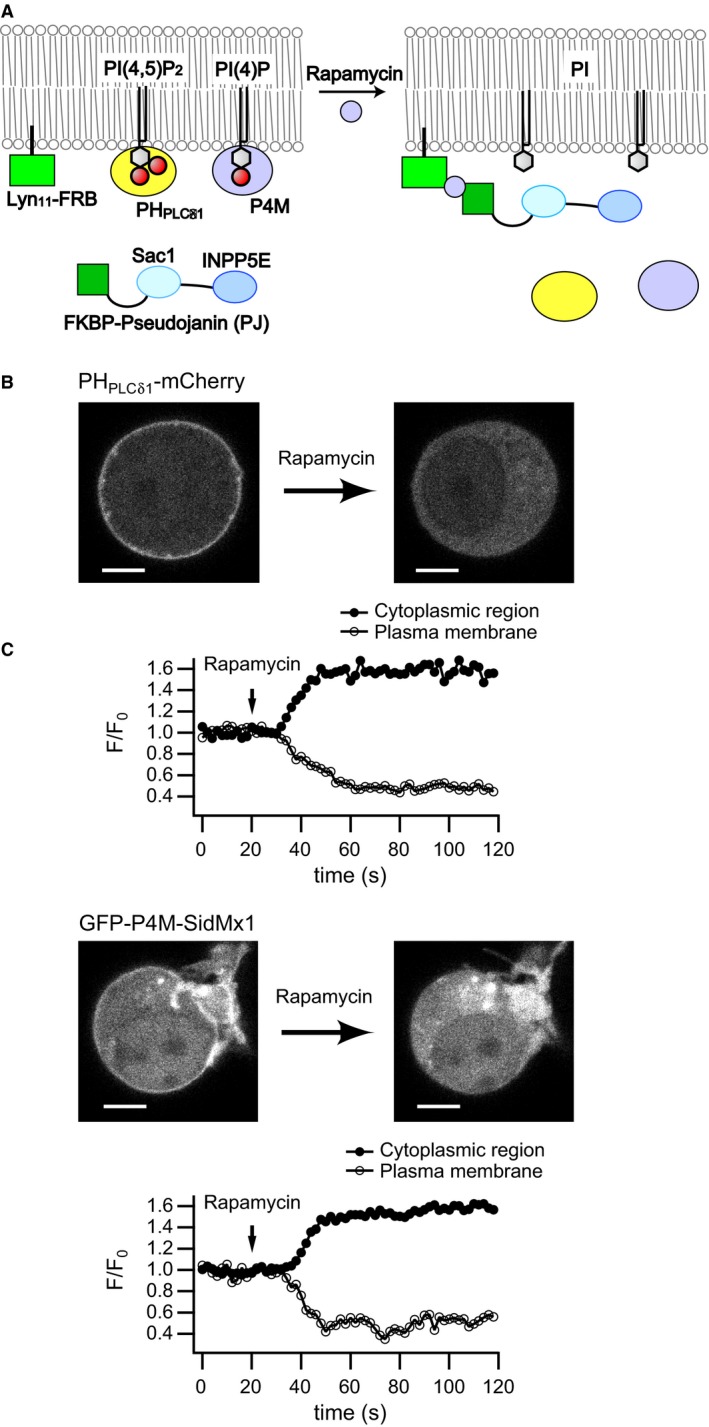
Changes in plasma membrane PI(4,5)P_2_ and PI(4)P upon activation of PJ in Neuro 2a cells. (A) schematic representation of PI(4,5)P_2_ and PI(4)P depletion upon PJ recruitment to the plasma membrane. PI(4,5)P_2_ and PI(4)P were monitored using PH_PLC__*δ*1_‐mCherry and GFP‐P4M‐SidMx1, respectively. After PI(4,5)P_2_ or PI(4)P depletion by PJ, these fluorescent probes translocate to the cytoplasmic region. Sac 1 and INPP5E are 4‐phosphatase and 5‐phosphatase, respectively. INPP5E, inositol polyphosphate‐5‐phosphatase E; PI, phosphatidylinositol. (B) Fluorescence images of PH_PLC__*δ*1_‐mCherry before (left) and after (right) application of rapamycin (upper). Representative time course of normalized PH_PLC__*δ*1_‐mCherry fluorescence intensity in the plasma membrane and cytoplasmic region (lower). Images were captured at 0 sec (left) and 118 sec (right) from a Neuro 2a cell expressing PH_PLC__*δ*1_‐mCherry, PJ, and Lyn_11_‐FRB. Scale bar, 5.0 *μ*m. Rapamycin (10 *μ*mol/L) was applied to the recording chamber at the time indicated by the arrow in the lower panel. (C) Fluorescence images of GFP‐P4M‐SidMx1 (upper) and representative time course of normalized GFP‐P4M‐SidMx1 fluorescence intensity in each region (lower). Neuro 2a cells expressing GFP‐P4M‐SidMx1, PJ, and Lyn_11_‐FRB were studied. Images were captured at 0 sec (left) and 118 sec (right). Scale bar, 5.0 *μ*m. Rapamycin was applied at the time indicted by the arrow in the lower panel.

We coexpressed PJ in Neuro 2a cells with PH_PLC*δ*1_‐mCherry, a PI(4,5)P_2_ sensor, or with GFP‐P4M‐SidMx1, a PI(4)P‐sensitive fluorescent probe (Hammond et al. 2014), and used confocal microscopy to assess the phosphatase activities of PJ (Fig. 5A). PH_PLC*δ*1_‐mCherry and GFP‐P4M‐SidMx1 moved from the plasma membrane to the cytoplasm after addition of rapamycin (Fig. 5B and C), indicating that both PI(4,5)P_2_ and PI(4)P were depleted in the plasma membrane. This experiment was performed in two more cells for each probe and similar results were obtained.

**Figure 6:**
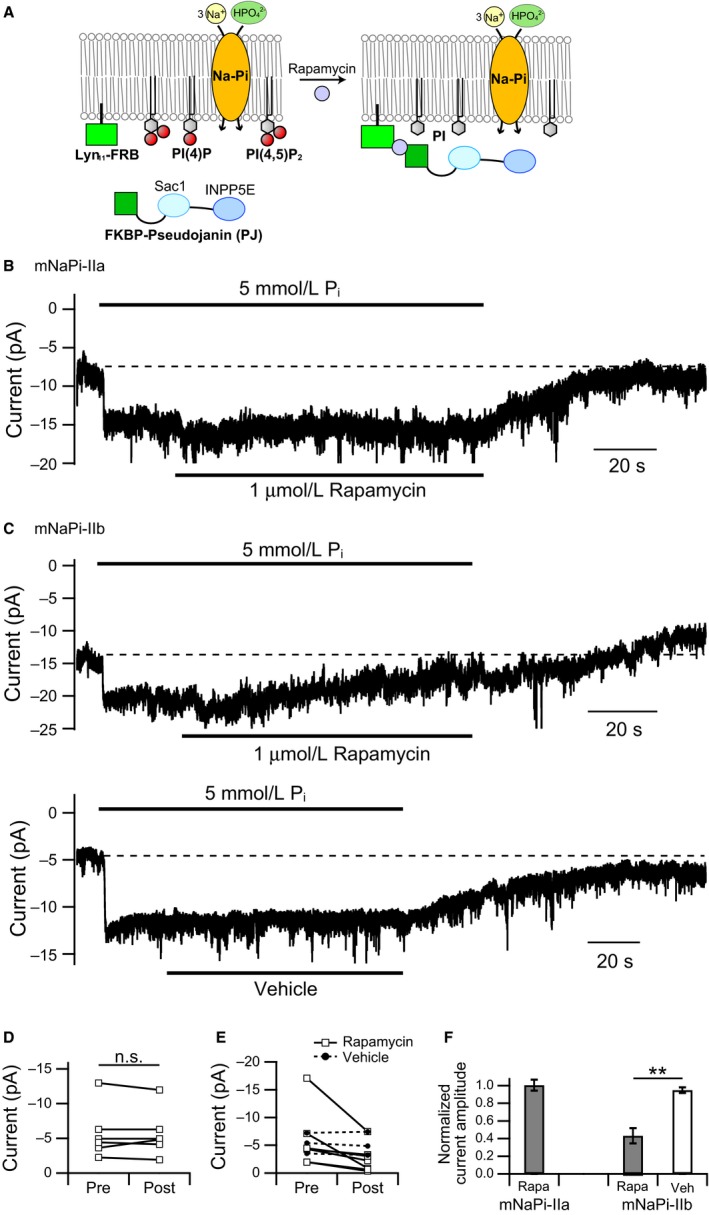
Effect of PJ‐induced depletion of PI(4,5)P_2_ and PI(4)P on NaPi‐IIa and NaPi‐IIb activities in Neuro 2a cells. (A) Schematic representation of the measurement of Na‐Pi cotransporter activity with PJ‐induced depletion of PI(4,5)P_2_ and PI(4)P. Rapamycin links PJ to Lyn_11_‐FRB present in the plasma membrane. (B) Representative current trace recorded from a Neuro 2a cell coexpressing mNaPi‐IIa with PJ and Lyn_11_‐FRB. Rapamycin (1 *μ*mol/L) was applied after P_i_‐induced transporter currents emerged. (C) Representative mNaPi‐IIb currents recorded from Neuro 2a cells coexpressing PJ and Lyn_11_‐FRB in the presence (upper) or absence (lower) of 1 *μ*mol/L rapamycin. In B and C, membrane potential was clamped at −60 mV and dashed lines indicate the initial current level before perfusing P_i_‐containing solution. (D) Comparison of the amplitudes of mNaPi‐IIa currents recorded before (pre) and after (post) perfusion of rapamycin. (E) Changes in mNaPi‐IIb current amplitude elicited by perfusion of rapamycin (open squares and solid line) or vehicle (filled circles and dashed line). (F) Summary of the effect of PJ‐induced depletion of PI(4,5)P_2_ and PI(4)P on mNaPi‐IIa and mNaPi‐IIb currents. Current amplitudes after rapamycin application were normalized to the amplitudes before application. Normalized amplitudes were compared using Student's *t*‐test (*n* = 6 for mNaPi‐IIa, *n* = 7 for rapamycin‐treated mNaPi‐IIb, *n* = 3 for vehicle‐treated mNaPi‐IIb, means ± SEM, **P <* *0.01).

With this system, we next examined the effects of PI(4,5)P_2_ and PI(4)P depletion on mNaPi‐IIa activity using whole‐cell patch clamp (Fig. 6A). Rapamycin was added after inward currents were induced by P_i_. mNaPi‐IIa currents were unaffected by the addition of rapamycin (Pre: 5.8 ± 1.4 pA, Post: 5.7 ± 1.3 pA, *n* = 6, paired *t*‐test: *P* = 0.80, ratio: 1.01 ± 0.06) (Fig. 6B, D, and F). By contrast, mNaPi‐IIb currents were significantly decreased after perfusion of rapamycin (Pre: 5.9 ± 1.8 pA, Post: 2.6 ± 0.9 pA, *n* = 7, paired *t*‐test: *P* = 0.037, ratio: 0.43 ± 0.09) (Fig. 6C upper panel, E, and F), but were unaffected by perfusion of vehicle (DMSO) (Pre: 5.4 ± 0.9 pA, Post: 5.2 ± 1.0 pA, *n* = 3, paired *t*‐test: *P* = 0.43, ratio: 0.95 ± 0.03) (Fig. 6C lower panel, E, and F). These results are consistent with the results from *Xenopus* oocytes and indicate that whereas mNaPi‐IIa activity is insensitive to the membrane PI(4,5)P_2_ level, mNaPi‐IIb activity is reduced upon depletion of PI(4,5)P_2_. We sometimes observed a decrease in the basal current after rapamycin application (Fig. 6C upper panel); that is, after washout of P_i_, mNaPi‐IIb currents decreased to a level lower than the initial current level before perfusing P_i_‐containing solution (Fig. 6C upper panel, dashed line). This phenomenon was not observed with vehicle application nor with mNaPi‐IIa (Fig. 6B and C lower panel). This suggests that both P_i_‐coupled current and the P_i_‐independent basal inward current of mNaPi‐IIb depend on PI(4,5)P_2_, as was suggested by experiments with oocytes (Fig. 3B).

## Discussion

In this study, we used voltage‐induced depletion of PI(4,5)P_2_ with Ci‐VSP and rapamycin‐induced depletion of both PI(4,5)P_2_ and PI(4)P with a PJ system to examine the sensitivity to phosphoinositides of two mouse electrogenic Na‐Pi cotransporters, mNaPi‐IIa and mNaPi‐IIb. Our results indicate that mNaPi‐IIa is insensitive to both PI(4,5)P_2_ and PI(4)P, whereas mNaPi‐IIb is sensitive to depletion of PI(4,5)P_2_. Our study also provided the first case of characterization of NaPi‐IIa and NaPi‐IIb by whole‐cell patch clamp recording. We found that these transporters exhibit a P_i_‐independent basal inward current and that a slow recovery after washout of P_i_ could be an innate property of both mNaPi‐IIa and mNaPi‐IIb. We also found that these transporters have distinct kinetics in response to application of P_i_. Collectively, our findings indicate that mNaPi‐IIa and mNaPi‐IIb have distinct functional properties including different sensitivities to PI(4,5)P_2_.

### Depletion of PI(4,5)P_2_ by VSP or Pseudojanin

VSP's nature of rapid and reversible PI(4,5)P_2_ depletion is effective for examining the PI(4,5)P_2_ sensitivity of ion channels and transporters, as it minimizes cell damage and enables repeated recordings from the same cell. With the PJ system, mNaPi‐IIb currents were also decreased during application of rapamycin (Fig. 6C upper panel). However, the rate of PI(4,5)P_2_ depletion was slower than with Ci‐VSP, and the depletion was irreversible because after combining with the scaffold protein (Lyn_11_‐FRB) through rapamycin, the phosphatase remains irreversibly anchored to the plasma membrane.

Insensitivity of any given protein to depletion of PI(4,5)P_2_ by VSP does not necessarily mean that the protein is totally insensitive to phosphoinositide, since dephosphorylation of PI(4,5)P_2_ by VSP produces PI(4)P (Iwasaki et al. 2008). For example, the activity of mammalian transient receptor potential vanilloid 1 (TRPV1) is unaffected by VSP‐induced depletion of PI(4,5)P_2_ (Lukacs et al. 2013), but its activity is suppressed by depletion of both PI(4,5)P_2_ and PI(4)P (Hammond et al. 2012; Lukacs et al. 2013), suggesting that mammalian TRPV1 is sensitive both to PI(4,5)P_2_ and PI(4)P. In our study, mNaPi‐IIa activity was not affected by Ci‐VSP‐induced PI(4,5)P_2_ depletion (Fig. 2B) or by depletion caused by the PJ system (Fig. 6B), indicating that mNaPi‐IIa is insensitive to both PI(4,5)P_2_ and PI(4)P. Thus, the combination of VSP and PJ enables precise evaluation of ion channel and transporter sensitivity to PI(4,5)P_2_ and PI(4)P.

### Regulation of NaPi‐IIb activity by PI(4,5)P_2_

During PI(4,5)P_2_ depletion by Ci‐VSP, mNaPi‐IIb activity was reduced to about 60% of the maximal current magnitude (Fig. 3E). Upon PI(4,5)P_2_ depletion by PJ (Fig. 6F), mNaPi‐IIb activity was still about 40% of the original current level. PJ depletes plasma membrane PI(4,5)P_2_ almost completely because of its dual enzyme activity (5‐phosphatase and 4‐phosphatase). These findings suggest that PI(4,5)P_2_ is not essential for mNaPi‐IIb activity but is necessary for full activity of mNaPi‐IIb.

How does PI(4,5)P_2_ bind to mNaPi‐IIb to regulate its transport activity? PI(4,5)P_2_ is known to bind to ion channels electrostatically (Hille et al. 2015; Dickson and Hille 2019). For example, X‐ray crystallographic analysis of the PI(4,5)P_2_‐binding sites on the inwardly rectifying potassium channel 2.2 (Kir2.2) showed that positively charged amino acids are important for PI(4,5)P_2_ binding (Hansen et al. 2011). By analogy, positively charged amino acids on the intracellular side of mNaPi‐IIb may be important for PI(4,5)P_2_ binding. A topological model based on epitope labeling, cysteine scanning mutagenesis, and in vitro glycosylation assays (Radanovic et al. 2006) showed that the N‐ and C‐terminal regions of type II Na‐Pi cotransporters are situated on the intracellular side of the protein and that there are five intracellular linkers. In mNaPi‐IIb, these intracellular regions contain many positively charged amino acids, which could be candidate sites for PI(4,5)P_2_ binding. Amino acid sequence alignment among type II Na‐Pi cotransporters shows that as compared to mNaPi‐IIb, a large section has been deleted from the C‐terminal region of mNaPi‐IIa and mNaPi‐IIc. This suggests that the C‐terminal region of mNaPi‐IIb may be important for its PI(4,5)P_2_ sensitivity. Several positively charged amino acids within the C‐terminal region of NaPi‐IIb conserved among mammals may be important for PI(4,5)P_2_ binding.

Both basal inward currents in the absence of extracellular P_i_ and P_i_‐coupled currents were reduced upon depletion of PI(4,5)P_2_ (Figs. 3B and 6C upper panel). Basal inward currents reflect a Na^+^‐dependent P_i_ transport mode intrinsic to electrogenic Na‐Pi cotransporters (Andrini et al. 2008; Forster 2019). Our finding that these two transport modes are sensitive to PI(4,5)P_2_ in mNaPi‐IIb suggests that they involve common structural changes, consistent with a previously proposed idea (Forster 2019).

Type II Na‐Pi cotransporters are conserved among species from bacteria to humans (Werner and Kinne 2001). With the exception of chickens and mammals, all tested species express only a single type II Na‐Pi cotransporter classified as NaPi‐IIb (Forster et al. 2011). NaPi‐IIa and NaPi‐IIc are reported only in chicken, mouse, rat, and human (Forster et al. 2011). Interestingly, the NaPi‐IIb homolog in winter flounder is found in tissues throughout the animal, including the kidney (Werner et al. 1994), but mammalian NaPi‐IIb is not expressed in kidney, where NaPi‐IIa and NaPi‐IIc mediate P_i_ reabsorption (Forster et al. 2011). These observations suggest that NaPi‐IIb is more closely related to the ancestor of type II Na‐Pi cotransporters than the other two isoforms are. It will be intriguing to know whether the teleost NaPi‐IIb homolog is PI(4,5)P_2_‐dependent. It is possible that PI(4,5)P_2_‐independence of mNaPi‐IIa is a more recent innovation along vertebrate evolution.

### Insights into physiological significance of different PI(4,5)P_2_ dependence between NaPi‐IIa and NaPi‐IIb

**Figure 7:**
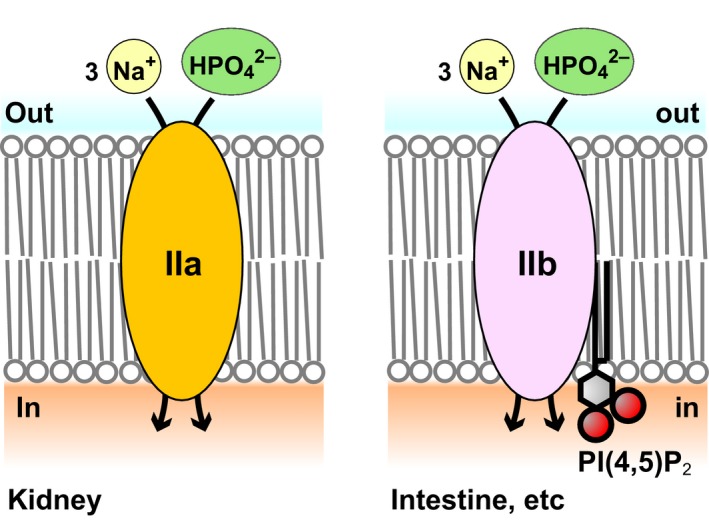
Conceptual model showing the different PI(4,5)P_2_ dependences of the electrogenic NaPi‐IIa and NaPi‐IIb cotransporters. NaPi‐IIa and NaPi‐IIb transport one HPO_4_^2−^ with three Na^+^. NaPi‐IIb activity, for example in the intestine, is regulated by PI(4,5)P_2_ in the plasma membrane. NaPi‐IIa activity in the kidney is unaffected by PI(4,5)P_2_.

Our finding that mNaPi‐IIa is insensitive to PI(4,5)P_2_ suggests that it does not require PI(4,5)P_2_ for its transport activity in renal proximal tubules (Fig. 7). The activities of many ion channels sensitive to PI(4,5)P_2_ are known to be altered by G_q_‐mediated activation of PLC, which hydrolyzes PI(4,5)P_2_ (Hille et al. 2015). In the kidney, G_q_‐coupled receptors such as PTH receptors are present on the basolateral membrane of epithelial cells in the renal cortical tubules (Amizuka et al. 1997). However, our results exclude the possibility that NaPi‐IIa activity is altered through changes of PI(4,5)P_2_ concentration upon activation of G_q_‐coupled receptors. It is possible that PI(4,5)P_2_‐independence of NaPi‐IIa activity is important for P_i_ reabsorption activity in the plasma membrane of renal proximal tubule cells. We also showed that mNaPi‐IIa is still active upon depletion of both PI(4,5)P_2_ and PI(4)P (Fig. 6B), raising a possibility that P_i_ transport activity could be maintained by NaPi‐IIa which is located in endomembranes such as endosomes or lysosomes. The transport ability of NaPi‐IIa within endomembranes may be inhibited by the acidic luminal environment (Demaurex 2002) for its pH‐sensitivity (de la Horra et al. 2000). In contrast to NaPi‐IIa, the activity of NaPi‐IIb in endomembranes, which have little PI(4,5)P_2_, may be limited due to its PI(4,5)P_2_ dependence.

Because G_q_‐coupled receptor signaling is the main pathway to PI(4,5)P_2_ hydrolysis, the same pathway likely regulates the activity of PI(4,5)P_2_‐sensitive NaPi‐IIb in cells that natively express it. NaPi‐IIb is known to be expressed at the apical pole of alveolar type II (AT2) epithelial cells (Traebert et al. 1999), which produce pulmonary surfactant. P_i_ is an essential component of surfactant, and NaPi‐IIb may bind P_i_ in the liquid covering the surface of the alveolar epithelium and provide it to AT2 cells. GPR116, a G_q_‐coupled receptor, reportedly localizes at the apical side of AT2 cells (Brown et al. 2017) where it negatively regulates surfactant secretion (Brown et al. 2017). This suggests that NaPi‐IIb's transport activity is inhibited by activation of GPR116. Thus, simultaneous restriction of P_i_ transport and surfactant synthesis through inhibition of NaPi‐IIb activity via activation of GPR116 may take place under some physiological condition.

NaPi‐IIb is also known to be expressed in intestine (Hilfiker et al. 1998). Our finding that NaPi‐IIb is sensitive to PI(4,5)P_2_ (Fig. 7) raises the possibility that an as yet unidentified G_q_‐coupled receptor signaling pathway may be important for P_i_ homeostasis in the intestine. The composition of ingested food is known to stimulate G_q_‐coupled receptors expressed by some types of secretory cells of the intestine (Reimann et al. 2012). This suggests the possibility that NaPi‐IIb may be involved in mediating mucus secretion, though it is not known whether NaPi‐IIb is expressed in mucus‐secreting cells.

The physiological significance of different PI(4,5)P_2_ sensitivity between NaPi‐IIa and NaPi‐IIb will be an interesting topic for future study.

### Kinetic properties of the two electrogenic Na‐Pi cotransporters, NaPi‐IIa and NaPi‐IIb

The slow recovery of mNaPi‐IIa and mNaPi‐IIb after P_i_ washout has previously been shown (Forster et al. 2002; Andrini et al. 2008). In these studies using *Xenopus* oocytes, the possibility of inefficient washout of external P_i_ could not be excluded. In the present study, mNaPi‐IIa and mNaPi‐IIb currents from cultured Neuro 2a cells under whole‐cell patch clamp using a rapid perfusion system also revealed the slow recovery after P_i_ washout (Fig. 4C and E). To test if the slow recovery observed in this study could be an innate property of these transporters, more rigorous experiments are necessary.

In this study, we succeeded in measuring electrogenic type II Na‐Pi cotransporter currents by whole‐cell patch recording for the first time. In the presence of P_i_, mNaPi‐IIb exhibited marked current decay after the current reached its maximum (Fig. 4E arrow), whereas mNaPi‐IIa did not show such current decay. Further experiments will be necessary to determine molecular basis underlying distinct kinetic properties between mNaPi‐IIa and mNaPi‐IIb.

## Conflict of Interest

The authors declare that they have no competing interests.
